# Implementing Cognitive Behavioral Therapy in the real world: A case study of two mental health centers

**DOI:** 10.1186/1748-5908-3-14

**Published:** 2008-02-29

**Authors:** Teresa L Kramer, Barbara J Burns

**Affiliations:** 1 Department of Psychiatry, University of Arkansas for Medical Sciences, Little Rock, AR, USA; 2 Department of Psychiatry and Behavioral Sciences, Duke University School of Medicine, Durham, NC, USA

## Abstract

**Background:**

Behavioral health services for children and adolescents in the U.S. are lacking in accessibility, availability and quality. Evidence-based interventions for emotional and behavioral disorders can improve quality, yet few studies have systematically examined their implementation in routine care settings.

**Methods:**

Using quantitative and qualitative data, we evaluated a multi-faceted implementation strategy to implement cognitive-behavioral therapy (CBT) for depressed adolescents into two publicly-funded mental healthcare centers. Extent of implementation during the study's duration and variables influencing implementation were explored.

**Results:**

Of the 35 clinicians eligible to participate, 25 (71%) were randomized into intervention (n = 11) or usual care (n = 14). Nine intervention clinicians completed the CBT training. Sixteen adolescents were enrolled in CBT with six of the intervention clinicians; half of these received at least six CBT manually-based sessions. Multiple barriers to CBT adoption and sustained use were identified by clinicians in qualitative interviews.

**Conclusion:**

Strategies to implement evidence-based interventions into routine clinical settings should include multi-method, pre-implementation assessments of the clinical environment and address multiple barriers to initial uptake as well as long-term sustainability.

## Background

Policy debates at the national level suggest that critical gaps exist in behavioral health services for children and adolescents in this country [1-3]. One approach to correct deficiencies in care is widespread implementation of evidence-based practices (EBP) such as those outlined in recently published reviews [4-7], practice guidelines [8-10], and other consensus documents [11]. Despite the inherent logic of this solution and advocacy by multiple stakeholders, adoption of scientific knowledge into routine practice remains limited and is one of the greatest challenges for policy advocates, funding agencies, and mental health administrators.

Much of the research on science-to-practice models has used diffusion theory to describe and examine variables associated with innovation adoption [12]. Rogers posited five stages to adoption: knowledge acquisition, persuasion, decision-making, implementation, and confirmation. Rate of adoption at the individual level is influenced by the perceived attributes of the innovation, as well as the type of innovation decision, communication medium, nature of the social system, and role of the change agent. Adoption at early versus later stages also depends on characteristics of the individual. According to Rogers, "innovators" – individuals who adopt an innovation in the very early stages – are more likely to control financial resources to absorb possible losses from an unprofitable innovation, possess an ability to understand and apply complex technical knowledge, and be able to cope with a high degree of uncertainty about an innovation. By comparison, "laggards" – individuals who are unlikely to adopt until very late in the diffusion process – are more likely to be isolated from their social network, conventional in their thinking and suspicious of innovations.

Similar to adoption by individuals, organizational innovativeness follows a linear path from initiation to implementation, and is associated with leadership and internal and external characteristics of the organization [13]. Multiple investigators have described similar models of innovation adoption in health care [14-18], as described further below.

### Organizational innovation

Although investigators have studied the implementation of EBP in health care, only a few have systematically assessed organizational variables that hinder or facilitate this process, and findings are mixed. At least two studies found no association between organizational culture *per se*, and adoption of a particular EBP [19,20]. Other investigators have determined that certain organizational characteristics (*e.g*., size, professionalism, leadership, quality improvement efforts, commitment, maturity, and resources) and change strategies that have an organizational component do influence the adoption of innovative practices [21-28].

### Clinician innovation

The literature on clinician variables associated with EBP implementation suggests that innovators and early adopters are more "tuned-in" and less provincial [29], more enthusiastic and organized [25], better educated [30], and involved early in the planning of an implementation strategy [31]. By comparison, provider characteristics associated with non-adoption of practices consistent with evidence-based guidelines may include lack of awareness and familiarity with guidelines, disagreement with guidelines, lower perceived self-efficacy and outcome expectancy for implementation of guidelines, and inertia of previous practice [32].

### Consumer innovation

Although consumers are central to the process of EBP use, there have been only a few studies examining how characteristics or preferences of this group may facilitate or hinder the adoption process. Non-compliance, refusal, or decreased opportunities for care due to not maintaining appointments are the primary consumer variables studied in this context [33]. Unfortunately, minimal attention has been devoted to the marketing aspects of intervention development and dissemination, leaving the field uninformed about patient preferences for specific treatments [34].

### Innovation in mental health services for youth

Despite progress in defining the parameters of EBP implementation in mental health care, only a few published studies have examined specific variables influencing successful or failed adoption of EBP for youth. For example, Schoenwald *et al*. [35] found that organizational climate and structure were generally unrelated to clinician adherence to multi-systemic therapy (MST) in real world settings. However, other investigators have found that characteristics of the innovator, clinician, and administrator are critical for the successful adoption of EBP [36-38]. In a recent study of a clinical intervention for juvenile firesetters, innovation characteristics were more salient in the early adoption phase, while adoptive and dissemination characteristics were more influential in actual implementation, suggesting that different factors are important at different stages [39]. More recently, Aarons [30] identified four factors contributing to adoption of mental health interventions: Requirements (an individual's willingness to adopt an intervention if required by their agency or related organization); Appeal (the extent to which an individual adopts an intervention if it is intuitively appealing, makes sense, could be used correctly or is being used by colleagues who are happy with it); Openness (the extent to which an individual is willing to try or use new interventions); and Divergence (the extent to which an individual perceives research-based interventions as not clinically useful).

Although organizational, clinician, and consumer variables constitute important factors in the implementation process, an important component often overlooked is the facilitation strategy. Kitson *et al*. [40] propose that successful implementation of EBP within an organization occurs when the evidence (research, clinical expertise, and patient preferences) is strong; the context (culture, leadership, and measurement) is receptive to change; and facilitation (skills of the change agent) is highly consistent. Thus, researchers as external facilitators of change play a key role in the organization's uptake of EBP.

The purpose of the present study was to explore implementation of cognitive behavioral therapy (CBT) for depressed adolescents seeking public sector mental health services. CBT was selected for study because it meets several of Rogers'[13] innovation criteria essential for diffusion: relative advantage, trialability, and compatibility. More specifically, CBT has been identified as an effective treatment for depression in adolescents [7]. When adopted in community settings, clinical outcomes of depressed adolescents at the 12-month follow up are superior to those of usual care [41,42]. Secondly, standardized treatment components and manuals for CBT have been developed to aid in the implementation process [43]. Finally, the theoretical background of CBT is also included in most graduate program curricula for psychologists, social workers and other mental health professionals, providing a familiar framework for dissemination to adolescent care [44].

In this study we investigated 1) the extent to which CBT for depressed adolescents was implemented in two publicly-funded mental healthcare clinics; 2) the process of CBT implementation in such settings; and 3) the factors influencing successful implementation of CBT, as cited by clinicians in monthly supervision sessions and in post-study qualitative interviews. The facilitation process consisted of initial discussion with clinic leaders and therapists about the treatment of depressed adolescents in their settings and the need for EBP. Facilitation by the research team also included training, supervision, and telephone reminders. A formative evaluation with input from manages and clinicians further guided the implementation process as it unfolded. Finally, summative evaluation, consisting of medical record review and qualitative interviews, was conducted to determine the extent to which CBT was implemented (compared to usual care) and the factors contributing to partial or full implementation.

## Methods

### Participants

Two urban mental health centers participated in this study. Center A is primarily publicly funded, with total revenue of $15 million. The full-time equivalent (FTE) is 57 for mental health professionals devoted solely to children's services. Center A serves approximately 800 unduplicated youth; one-fourth of these are in school-based settings. Seventy percent are male. Center B is also primarily publicly funded with a slightly larger revenue ($22 million) and 45 FTE devoted to mental health professionals for children's services. Center B serves approximately 1600 unduplicated youth; one-third of these are in school-based settings. Fifty-four percent are male.

Clinicians were eligible to participate if they anticipated providing therapy to at least two depressed adolescents (ages 11–18) in an outpatient or school-based setting per month during the course of the study, which was anticipated to extend for at least one year. Of the full-time clinicians in Center A, 17 were eligible; nine agreed to participate. Of the full-time clinicians in Center B, 18 were eligible; 16 agreed to participate. Full-time clinicians were expected to bill 24–26 hours per week, depending on their other supervisory or administrative responsibilities. No credit toward productivity was allotted for cancellations, no shows, or training. Actual caseloads varied from 35 to 60 clients.

### Formative evaluation

Qualitative and quantitative data were collected to inform the implementation process. We initially discussed with the medical director and quality improvement manager of each center issues relevant to the feasibility and acceptability of the research, including: 1) data security, consent procedures, and other measures to assure adolescent/parent confidentiality and compliance with Health Insurance Portability and Accountability Act [45]; 2) duration, location, and other logistics of CBT training and supervision; 3) screening and referral procedures for eligible adolescents; 4) compensation for clinician participation outside the scope of their usual duties; and 5) procedures for medical record review and audiotaping of sessions. During these initial stages of the formative evaluation, we collected specific information pertaining to "how-to" knowledge [13] including 1) preference for a one-day training followed by monthly supervision of participating clinicians, 2) approval of a brief, nine-session CBT intervention that combined psychosocial intervention, medication monitoring and motivational interviewing [43]; 3) tools and mechanics for screening depressed youth in the clinics; and 4) staffing needs and resources to conduct screening and complete paperwork necessary for the study itself. The clinic managers identified a psychologist in each clinic who did not participate in the intervention but received $5,000 in salary support from the research team to monitor enrollment and screening of adolescents, collect the CDI when completed by adolescents, follow-up with clinicians to identify problems in recruitment, communicate study concerns to the research team, facilitate audiotaping and medical record review, and organize supervision sessions.

Clinicians also completed the Provider Attitude Survey, a modified version of the questionnaire developed by Addis & Krasnow [46], based on research on EBP implementation conducted by Cabana *et al*. [32]. The 27-item survey assesses clinician knowledge, awareness and attitudes toward CBT and manualized treatments in general; current practices using CBT; and intentions to initiate CBT in the next six months. At the close of the CBT training, clinicians also completed a nine-item survey to assess satisfaction with the instruction, attitude toward CBT and level of comfort with CBT initiation. Investigators also collected qualitative data through field notes of discussions with managers and records of supervisory sessions with intervention clinicians. Results from the surveys and ongoing field notes were used to further facilitate EBP implementation, for example, refining the content of supervision sessions with clinicians, clarifying CBT questions for clinicians on an ongoing basis, addressing system-wide barriers to screening and recruitment, and minimizing the effects of attrition on the overall study design.

### Summative evaluation

Key informant interviews were conducted at the close of the study with all intervention clinicians, two clinical managers at each clinic, four clinicians providing usual care, and three clinicians who dropped out prior to formal consenting. Interviews, which lasted approximately 30 to 45 minutes, were audiotaped, transcribed, reviewed for accuracy, and entered in Ethnograph for data management purposes.

In order to assess the extent to which CBT was provided, trained research assistants reviewed all sessions in each adolescent's medical record to determine whether CBT was mentioned as the primary treatment. Research assistants were trained to assess whether CBT components consistent with the manual were documented for no sessions, one to three sessions, four to six sessions, or more than six sessions, with 80% concordance. Because of the self-report nature of medical records, we also randomly collected audiotapes of sessions from five clinicians. Three clinicians did not provide any audiotapes for review (two of whom did not enroll any adolescents in the study). These were reviewed by the first author who was blind to the medical record review for the presence of CBT components in the manual, including cognitive restructuring, mood monitoring, completion of a pleasant events checklist, behavioral contracting for pleasant events, or discussion of home exercises. Audiotapes were rated as to whether the clinicians engaged in CBT with the adolescent during that particular session. Concordance rate between the audiotape and medical record review for that session was 100%.

### Procedures

Based on data generated from early stages of the formative evaluation, we developed and submitted a study protocol to the UAMS Institutional Review Board (#15068; approved 16 October 2002). Oversight committees for research at each of the sites also reviewed and recommended modifications to the protocol before the study began. Because these were off-campus clinics engaging in research, we also obtained site authorizations to conduct research for each.

Clinician recruitment occurred at a regularly scheduled staff meeting at each clinic during which the first author presented data regarding usual care established through a previous study [47], evidence on the effectiveness of CBT with depressed adolescents, and information on the proposed study. Follow-up phone calls were initiated for all eligible clinicians, including those who did not attend the staff meeting. Following a formal consenting process, clinicians were randomized into CBT training versus usual care.

Training consisted of instruction in: components of motivational interviewing to engage the adolescent in CBT; educating the adolescent about depression; ongoing assessment of suicidal risk and, if applicable, medication adherence; and CBT (four sessions of cognitive restructuring and four sessions of behavioral activation) [43]. Monthly CBT supervision augmented weekly supervision required for unlicensed clinicians (two hours per week), and monthly supervision required for licensed clinicians (one hour per month).

Screening of adolescents, ages 11–18 years, occurred at the initial visit by administrative personnel or the designated clinician, using a cut-off of 12 or above on the Children's Depression Inventory (CDI) [48]. The CDI is a 27-item self-report survey measuring depression severity on a scale of 0 to 54 with five subscales (negative mood, interpersonal problems, ineffectiveness, anhedonia, and negative self-esteem). Internal consistency and test-retest reliability are high for the measure; concurrent and discriminant validity are acceptable as well as sensitivity to changes in depression over time. Adolescent exclusion criteria included: imminent suicidal risk, severe conduct disorder, mental retardation, and referral for inpatient or residential treatment.

Eligible adolescents and their parents agreed in writing to provide their names and telephone numbers to the study team for telephone contact to explain the purposes of the study, screen for additional exclusion criteria (parental or adolescent cognitive impairment as evidenced during the telephone contact or in response to the question, "Does your son or daughter have any learning or other problems that might make it difficult to participate in this study?"), and initiate the formal consenting process. If parents and adolescents verbally agreed to participate, they received consent forms within three to five days by mail to sign and return. Signatures on the written consent form were required prior to data collection.

### Data analysis

Quantitative data analysis consisted of descriptive statistics for clinician demographics, CBT knowledge and attitudes, and post-training skills. Qualitative data derived from field and supervision notes were reviewed, and a code book developed for first-level coding. Raters were trained to achieve 80% concordance on first-level coding. Once first-level coding occurred, the first author developed a secondary coding scheme for the data, which is detailed in results.

The validity of the findings was addressed in several ways. First, we collected data from multiple sources, providing an opportunity to triangulate the data [49]. Triangulation may be particularly relevant to the examination of organizational culture, because different methods can be used to target different layers of culture [50]. Second, two research assistants coded the raw data and agreed on the coding scheme. Inter-rater concordance was established at 80% using four of the key informant interviews. The remaining data were coded independently with team meetings held regularly to resolve questions until consensus was established. Finally, the findings were presented to two groups of mental health services researchers and clinicians at both clinics to confirm accuracy of the final coding scheme and reasonableness of the findings.

## Results

Of the 35 eligible clinicians, 25 agreed to participate in the study and were randomized to intervention (n = 11) versus usual care (n = 14). However, of those randomized, 21 attended the introductory session, 18 completed consent forms and pre-intervention assessments, and 17 actually continued in the study. Thus, nine clinicians (three from Center A and six from Center B) completed the training, and eight clinicians (three from Center A and five from Center B) were assigned to usual care. All 18 consenting clinicians were female, with one exception. They all held at least a master's degree in social work (n = 10), counseling (n = 7), or psychology (n = 1). Two were African American; one was Asian American; and 15 were Caucasian. Non-participants included psychologists, social workers, and counselors; however, no other data were collected from these individuals.

Responses to the Provider Attitudes Survey prior to initiation of the study (n = 18) indicated that 14 clinicians (78%) had no experience with a treatment manual for CBT, 12 (66%) had no formal CBT training, and 16 (88%) had no prior CBT supervision. Twelve clinicians (66%) indicated they intended to use CBT treatment manuals sometimes, often, or always in their clinical practice in the next six months. Eight (44%) said they never or rarely used evidence-based or empirically-supported treatments for youth depression in clinical practice, and five (27%) said they planned to never or rarely use evidence-based or empirically-supported treatments for youth depression in the next six months. There were no differences between the two sites or clinicians assigned to the intervention versus usual care groups on any of these variables.

Training consisted of the rationale for using CBT in treating depression, session-by-session review of the manual, interactive discussions, role-playing, and exploration of barriers and strategies to assist in CBT implementation. Clinicians received continuing education credits for their participation in the training. Post-training surveys from intervention clinicians indicated that the majority understood the basics of CBT (62%), were aware of barriers that may occur in providing CBT in their settings (87%), and possessed a set of skills to address the barriers (95%). Although all clinicians indicated they had a positive attitude toward CBT, only one-half stated they felt prepared to implement CBT on a regular basis. In order to facilitate provision of CBT, they were asked to establish goals and monitor their implementation success following training, *e.g*., practice with an adolescent by the next monthly supervision meeting. Supervision was provided based on the case presentation of the clinicians and their stated needs (*e.g*., difficulty implementing CBT with adolescents in crisis).

During the study, 66 adolescents screened positive on the CDI, 49 agreed to be contacted by the research team, 39 were deemed eligible for the study, and 34 completed formal consents and assents (parents and adolescents, respectively). Sixteen were assigned to intervention clinicians. Twenty-one (62%) were female, and 22 (65%) were Caucasian; mean age was 13.5 years. Most were enrolled in either sixth (27%) or seventh grades (27%), although there were also adolescents in fifth grade (6%), eighth grade (12%), ninth grade (12%), tenth grade (6%) and eleventh grade (6%). A large majority (82%) lived at home with their parents; 6% lived with their adoptive parents; 6% lived with other relatives; 3% lived with friends; and 3% lived with someone other than the above. There were no significant differences on demographics or depression severity for adolescents assigned to intervention versus usual care conditions.

Following training during the adolescent enrollment stage, intervention clinicians were asked to initiate screening for depression, introduce the intervention to adolescents and parents, engage in manualized CBT, and participate in monthly supervision. At the close of the study, three (19%) of the charts indicated no provision of CBT, three (19%) of the charts indicated the clinician followed the CBT manual one to three sessions, two (12%) of the charts indicated the clinician followed the CBT manual four to six sessions, and eight (50%) of the charts indicated the clinician followed the CBT manual more than six sessions (see Table 1). Average number of therapy sessions was 16 (S.D. = 21.82). Three clinicians did not enroll any adolescents in the study; one clinician enrolled two adolescents in the study but did not provide CBT to either. There were no differences between clinicians who followed the manual for at least six sessions and clinicians who followed the manual fewer than six sessions on their prior training in CBT with adolescents. As expected, none of the adolescents in the usual care arm received CBT as determined by medical record review

**Table 1:** CBT implementation by clinician according to Medical Record Review (MRR), audiotape and interview

**Clinician**	**A**	**B**	**C**	**D**	**E**	**F**	**G**	**H**	**I**
Prior CBT Formal Training	---	---	X	---	---	X	X	---	---
# of Adolescents Enrolled in CBT Study	2	2	4	4	0	2	0	2	0
# of Adolescents Receiving >6 CBT Sessions (MRR)	0	1	2	2	0	1	0	2	0
# of Adolescents Receiving 4–5 CBT Sessions (MRR)	0	1	1	0	0	0	0	0	0
# of Adolescents Receiving 1–3 CBT Sessions (MRR)	0	0	0	2	0	1	0	0	0
# of Adolescents Receiving At Least 1 CBT Session (MRR)	0	2	3	4	0	2	0	2	0
# Number of Adolescents Receiving At Least One CBT Session (Audiotape)^a^	---	1	2	2	---	1	---	2	---
Clinician Reports Using CBT in Practice^b ^(Interview)	1	1	2	2	0	2	2	2	1

^a ^Number of audiotapes submitted: Clinician B = 1; Clinician C = 2; Clinician D = 2; Clinician F = 1; Clinician 2 = 2. ^b^0 = No adoption of CBT; 1 = Adoption of CBT components; 2 = Adoption of CBT intervention

Five of the six clinicians who enrolled adolescents in the study submitted an audiotape of at least one of their sessions. Results of the audiotapes indicated that five of the six clinicians provided at least one session of manualized CBT.

Although all nine clinicians participated in at least three sessions of monthly CBT supervision, attendance gradually declined, resulting in attendance by only one clinician from each clinic (both of whom engaged in CBT with high fidelity) by the end of the study. (Of note, one of the intervention clinicians had left the agency; another had been reassigned to residential care.)

During the qualitative interviews, eight of the nine clinicians in the intervention group reported that they continued to provide CBT for depressed adolescents in an outpatient or school-based setting. Of these, five reported they adhered consistently to the manual, while three reported they used "CBT components" adapted from the manual. Notably, as Table 1 indicates, there were three clinicians who did not use CBT in the study but reported in interviews that they were still using CBT. One clinician reported using CBT with adults, given that there were only a few adolescents on her caseload. Another clinician who did not enroll any adolescents in the study said she nonetheless has followed the manual with at least two adolescents.

Data derived from supervision notes and key informant interviews suggest that multiple inhibiting or activating variables at each phase contributed to or inhibited successful implementation of CBT. These were categorized into consumer (adolescent or parent), clinician, intervention, organization, and external environment characteristics, similar to the domains identified by Schoenwald and Hoagwood [51]. Examples from clinicians' qualitative interviews are delineated in Table 2 [see Additional file 1].

In seven of the nine interviews, intervention clinicians stated that productivity demands and recent changes in paperwork requirements by the clinic's primary payer had limited their ability to participate in the study and specifically engage in new learning. Eight of the nine intervention clinicians had difficulty due to the adolescent's cognitive deficits, family crises or co-morbid psychiatric problems. Five clinicians stated that their caseload changed during the course of the study so that they were not treating as many depressed adolescents as originally anticipated; these therapists were either seeing younger or behaviorally-disordered children. Other categories cited as problems by the majority of clinicians fell into the following categories: consumer (problems with adherence and acceptance), intervention (complexity), and provider (difficulties in coping with professional stressors). Four of the clinicians commented positively on the effectiveness of the intervention with the adolescents.

Clinicians who were able to adopt and sustain CBT reported they were able to balance between adolescent and family needs, deal effectively with clinical crises within the context of CBT, and adapt to external requirements and constraints, *e.g*., meeting productivity, completing paperwork, etc. Not only were they competent in their roles, but they displayed positive attitudes about the intervention from the initial to final stages of the project. They remarked, "I really enjoyed doing the CBT;" "I feel like I've learned a lot doing this;" and "I can now add this to my clinical repertoire." Of the clinicians who consistently provided CBT, none stated that organizational factors facilitated their adoption of the intervention.

## Discussion

This study demonstrated that CBT can be implemented to a moderate extent in publicly-funded mental health settings. Six of nine intervention clinicians enrolled adolescents in the study; five of these actually provided CBT to at least one adolescent. As a result, half of the adolescents presenting with depression received a significant "dose" of CBT (six or more sessions), which is significantly more than the adolescents enrolled in usual care. Thus, there was an improvement in the rates at which evidence-based care was provided in both centers. Moreover, all clinicians except one reported being more favorably inclined to include components of CBT in their work with adolescents due to their increased exposure to the intervention through training and supervision. Clinicians did not indicate in follow-up interviews that they perceived family therapy or psychodynamic therapy more effective than CBT, suggesting that CBT as an innovation had a certain attractiveness to participating clinicians. The more the pattern of benefits and risks of CBT "map" onto these interests and values, the more likely CBT will be adopted [52]. As clinicians decided to participate in training, ongoing supervision, and actual implementation, they appeared to be collecting personal evidence about the suitability and effectiveness of CBT for their clients, many of whom had co-morbid psychiatric and medical illness, chaotic lives, limited cognitive ability, and scarce resources – attributes that would usually preclude inclusion in randomized clinical trials.

In supervision and again during follow-up interviews, clinicians discussed their own limitations and biases, which interfered with their ability to become proficient at CBT. They acknowledged their difficulties in coping with the stress of their environment and admitted that they were too disorganized to learn a new intervention by enrolling adolescents in the study, reading through their training materials, or practicing on adolescents already on their caseloads. Clinicians who consistently provided the manualized CBT reported being excited about the intervention, confident of their skills, able to adapt the intervention to the needs of the adolescent and his or her family, and willing to continue to practice CBT beyond the confines of the study.

In addition to concerns about their own personal barriers as well as the appropriateness of CBT for their clients, clinicians also discussed problems at the level of the organization and external environment. Although leadership commitment was essential to introduce CBT into the centers, other factors, such as the organization's learning environment, determined clinicians' ongoing ability to enroll adolescents and engage in CBT. Thus, although leaders and clinicians were enthusiastic at the outset, implementation may have failed, in part, because ongoing supervision, collaboration with other clinicians, and "booster" training sessions were not adequately supported by the larger system. It is important to note that clinicians who did not consistently provide CBT described multiple organizational and environmental variables that diminished their ability to learn and apply CBT. They were more likely to blame their lack of implementation on paperwork, productivity requirements, and limited staffing support for screening. By comparison, clinicians who actively recruited and engaged in the study did not state that organizational or environmental factors facilitated their work. This finding suggests an interaction between activating and inhibiting variables at the clinician and organizational levels. When a motivated, competent clinician chooses to adopt an EBP, environmental factors may play a negligible role in the dissemination process. In contrast, clinicians with fewer skills or flexibility may need stronger organizational or environmental incentives to initiate or sustain such practices.

The findings also suggest that CBT implementation can be a complex, dynamic, and chaotic process. As noted by Redfern and Christian, implementation linearity is more apparent in organizations with high levels of certainty [53]. They suggested that organizations characterized by high turnover, inadequate staffing, or other disruptive conditions, as evidenced by the two centers in this study, may exhibit more disorganized patterns of implementation.

Findings from this study have numerous implications for practice. First, the results strongly suggest that successful dissemination of an EBP such as CBT requires assessment of the implementation culture at the level of the consumer, clinician, organization, and external environment as well as adaptation of the intervention to fit the target population. At the consumer level, strong consumer acceptance, engagement, and advocacy for CBT would have greatly enhanced the implementation efforts. Furthermore, CBT for adolescents may be more acceptable to parents when it is augmented with case management and/or family interventions that support systemic change as well as that of the adolescent. With regard to the CBT itself, training manuals and other dissemination tools must be created that allow for flexibility in the treatment process. Guidance should be provided on addressing co-morbid symptoms, particularly trauma, aggression, and substance use, and targeting adolescent resistance and non-adherence. In addition, EBP will not be effectively disseminated through manuals or toolkits alone. Often referred to as a "passive educational strategy" [54], this approach rarely results in behavioral change. Clinicians need external facilitation that includes in-depth training, ongoing supervision and technical assistance to acquire the "how-to" knowledge that will enhance their ability to overcome clinical issues, such as family or adolescent crises that interfere with a more structured approach to treatment or parent preferences to be more involved in the treatment process. Strategies to assess and improve clinician's innovativeness are also indicated. Measures developed to evaluate decision-making, such as the Kirton Adaption-Innovation Inventory [55,56] the Consumer Novelty Seeking/Consumer Independent Judgment Making Scale [57], or the EBP Attitude Scale [30] may provide a new direction for researchers in identifying and assisting clinicians who may have difficulty in adopting new interventions. At the organizational and external environment levels, the findings emphasize that clinicians need organizational support to cope with environmental threats. Often described as "resiliency," the organization must attain the capacity for continuous reconstruction to cope with changes in the external environment [58]. Public mental health systems, represented by the two clinics in this study, are particularly vulnerable to policy and fiscal changes and must therefore expend considerable effort to effectively implement and sustain EBP. For example, stringent productivity and paperwork requirements have the potential to compromise new learning, innovation, and creativity. In addition, when the external environment is stable and organizational climate is supportive – as opposed to more volatile circumstances – clinician innovativeness may play a less important role in EBP adoption. Organizational leaders may also want to select individuals who are positively inclined toward practicing EBPs and who are willing to supplement their previous experiences with additional training.

This study was limited by several weaknesses that may affect the interpretation of results. First, because data were collected from only two centers, the results may not generalize to other clinicians or clinics of varying size, staffing, and infrastructure. In addition, there were multiple environmental issues affecting clinician's workload that may not have relevance in other states, particularly with regard to changes in paperwork required by the primary payer, clinical processes, and financial solvency of the centers. Second, clinicians were sporadic in their adherence to the study methods, such as recruiting adolescents, providing audiotapes of sessions, or participating in regular supervision. Thus, our ability to confirm fidelity to the CBT model was limited. Although the qualitative interviews provided some data regarding clinician adoption of CBT, these were based on self-report and therefore may be biased toward providing a favorable impression for the research team. In addition, observation of a particular process necessitates the intrusion of a researcher and data collection that may, in effect, change the natural flow and outcome of the studied phenomenon. Thus, clinicians may have been less willing to participate because of their views about research or anticipation of more work in an already busy schedule. They may also have been more willing to adopt CBT, knowing that their success was being monitored. While community-based participatory research designs may mitigate these effects, the influence of the researcher as a change agent must nonetheless be noted, particularly when considering the sustainability of the intervention once the study ends. Finally, due to the small number of adolescent participants and limited power, it was not feasible to determine the effectiveness of CBT in reducing symptoms. Future implementation trials with multiple clinics and clinicians as well as a larger adolescent sample are warranted.

## Conclusion

In summary, this study illustrates the complexity of EBP implementation in routine care, particularly for psychosocial interventions that are not easily transported from the laboratory to the real world. Although treatments such as CBT show considerable promise for alleviating depression and preventing future episodes, multiple barriers – at the consumer, clinician, organizational and environmental levels – may prevent initiation and sustainability of such practices. Large, multi-site studies are needed to determine characteristics of clinicians and clinics that enable an EBT to be implemented and sustained, including clinician caseloads and productivity requirements as well as organizational resources. A critical component of future work should also include development of a set of data collection tools that will allow for succinct measurement of the pre-implementation environment, including leadership support, available resources, and clinician openness to EBP.

## Competing interests

The author(s) declare that they have no competing interests.

## Authors' contributions

TLK conceptualized, implemented, and conducted this study with consultation and guidance from BJB. Both TLK and BJB contributed to the writing of the manuscript. Both authors read and approved the final manuscript.

## Supplementary Material

Additional file 1 Variables influencing implementation of Cognitive-Behavioral Therapy (CBT). The table provides qualitative information from the clinicians about the variables contributing to the implementation of CBT. Click here for file
