# Supplementary material for: Implementing Cognitive Behavioral Therapy in the real world: A case study of two mental health centers
Source: Implement Sci. 2008 Feb 29;3:14. doi: 10.1186/1748-5908-3-14 (PMC2294138; doi:10.1186/1748-5908-3-14)
Supplement: Additional file 1 — Variables influencing implementation of Cognitive-Behavioral Therapy (CBT). The table provides qualitative information from the clinicians about the variables contributing to the implementation of CBT. [file 1748-5908-3-14-S1.doc]

**Table 2. Variables influencing implementation of Cognitive-Behavioral Therapy (CBT)**

| **CONSUMER** | **CLINICIAN COMMENT** |
| --- | --- |
| **□** Acceptance | “The ones for me that understood and accepted this (CBT) the best were the parents that were in treatment themselves, kind of the treatment-savvy parents.”  “Parents didn’t think that they were seeing enough improvement…maybe I didn’t do a good enough job explaining to the parents that, you know, it isn’t going to be instant improvement.”  “I saw kids I knew were depressed, and they put nothing (on the screening instrument).” |
| **□** Resources | “They don’t do anything pleasurable…not because they don’t want to, but they’re deprived. Most of them have single parents at poverty level in bad neighborhoods. They can’t even take a walk safely, so you know, I can’t say, ‘Oh, go out, have sunshine. No, they can get shot.’”  “They have to be able to call a friend or go skating, like I said in the beginning, and if you don’t have that, I mean one of the things I ran into is everything we looked at was not available to this child. That was depressing.” |
| **□** Availability | “You go to pull them out of class (for therapy) and the school says they’ve moved.”  “My kids are just unsteady, unstable, you know, they come and go.” |
| **□** Appropriateness | “It’s really hard in our situation to get a pretty pure depressed child. It’s usually multidiagnosed.”  “If they were cognitively, like low, like mild MR or something like that, I didn’t even use the book because they just lacked the insight.”  “There was always something that seemed to interfere with that (CBT)…got kicked out of school, got moved to foster home, no transportation.”  “I’m in the SED classrooms and there’s always something horrible going on…somebody getting arrested, somebody trying to jump on somebody else.”  “The biggest problems I see is that a lot of our kids seem to be in constant chaos or constant trauma just because they come from very dysfunctional families.” |
| **□** Adherence | “I don’t think I had one child complete homework that I asked.”  “I tried maybe a thing or two from the workbook, like gave the list of fun activities and asked her to pick a few things out of there, and she wouldn’t even do that.”  “So he knew he was depressed, he knew he had a problem, and he knew he didn’t want to live, so when I came along and really offered him my relationship, my willingness to help him, he was very motivated.” |
| **CLINICIAN** |  |
| **□** Openness to EBP and/or CBT | “I wanted to have that skill set under my belt, because I did come from that psychodynamic developmental and more relational kind of stuff, so I wanted something more concrete.”  “I think it (CBT) is great. I think they should teach CBT in high school curriculum.”  “Teenagers who are depressed and having behavior problems respond real well to having that kind of structure and having a level of information about what’s happening to them and how they can increase their control over their symptoms.”  “I read the literature and try and stay current on treatment…I don’t like feeling as though I’m ripping off (third-party payer) by continuing to keep kids in counseling when they’re not getting any real benefit or using interventions that don’t seem to help.” |
| **□** Caseload Mix | “My caseload was full and pretty much remained full, so I just didn’t take on any new clients that I could start (CBT) with.”  “I was given a lot of the younger kids and I wasn’t given the opportunity to do intakes with adolescents.” |
| **□** Coping Skills | “We were so stressed out by our day-to-day situations that we didn’t have any creative room.”  “For a while I felt like I was drowning.” |
| **□** Competence | “But I’m staying on top of the paperwork.”  “I feel comfortable with the fun activity part.”  “But it made me feel good to look at the workbook and say, ‘Okay, I do that, I do that, I do that, so you know it made me feel a little better.’” |
| **□** Learning Style | “It was helpful to hear…how they (other therapists) were doing and what obstacles that they had, so that I could learn from that.”  “I’m a learning-oriented kind of therapist.” |
| **INTERVENTION** |  |
| **□** Effectiveness | “I knew it was a very good tool for depression.”  “It makes sense as a treatment option.”  “The study reconfirmed for me how effective CBT can be.” |
| **□** Complexity | “The exercises and activities were just real plain and straightforward.”  “It was a little overly structured for my style, let’s put it that way, and I did a lot of improvisation.” |
| **□** Training/Tools | “I thought that was the perfect amount of time for the training.”  “I thought it was really helpful to do the role plays.”  “Maybe even a short video snip of this is what you do…that would have worked for me.”  “To improve (the manual), it might be color coding, with like a prime message with the most important part.” |
| **□** Compatibility | “When they (adolescents) see a manual and paperwork, it reminds them of classroom.”  “It’s almost too cognitive, too sophisticated.” |
| **□** Adaptability | “Those individual pieces are easily adaptable to any other kid that you’re working with.” |
| **ORGANIZATION** |  |
| **□** Learning Environment | “I don’t think there’s a lot of emphasis on evidence-based therapy...even with adolescents, there’s a lot of play therapy, nondirective sorts of interventions.”  “To have all these kids (on my caseload) and to stop just to come here for a three-hour training once a month, but we’re supposed to keep our productivity.”  “We’re very tight with our productivity requirements, and so anything that slows down the operation of the clinic, sometimes it can be very hurtful to therapists.”  “I have a heavy caseload (59 clients) and it’s a lot of time, you know, I would have trouble getting enough time to do prep about…what are we going to do next time.” |
| **□** Resources | “My stuff’s in the trunk of my car. I don’t have an office here (at school); I have a filing cabinet. We don’t have the facilities to keep notes and stuff.”  “There are times when I jump in the car and race off to (the clinic) and realize that I didn’t bring the game, the workbook…and I don’t have an office. I don’t have a filing cabinet; the principal asked me to remove it.” |
| **□** Target Population | “I get more of the destructive, aggressive acting out boys because that’s who the school’s saying, ‘Please, please help us, you know, take them first.’” |
| **□** Morale | “I think probably everybody just felt overloaded.”  “We were so overwhelmed in general…we didn’t have any creative room (to be more involved in the CBT implementation).” |
| **□** Retention | “But I’ve gone through so much transition. I’ve trained counselors and case managers who have come and then they go away.”  “Our agency went through such shifts in terms of management and in terms of, you know, loss of therapists, shifting of roles…We had therapists who had been there quite some time that were leaving that were frustrated.” |
| **□** Leadership | “Nobody (in the clinic) set forth the expectations.”  “I don’t really want recognition from my peers, but some sort of silent recognition from the boss: ‘I hear you’re doing this stuff (CBT implementation).’” |
| **EXTERNAL ENVIRONMENT** |  |
| □ Competing Requirements | “It (the paperwork) is excessive…I can tell you I’m not gonna stay in this job. Because it’s just ridiculous…I would start crying if I started to calculate it (the number of hours spent on paperwork.)”  “It (CBT implementation) happened at the same time when all of the (primary payer) things changed, when we had all new requirements to see kids, all new paperwork, all new everything.”  “I’m doing more paperwork than I’m actually spending face-to-face with the kids.” |
| □ Opportunities | “This year, I have not gotten one flyer or one brochure or anything like that on depression as a training to go to.”  “We asked about (CBT) in graduate school. They said, ‘You can learn that, you can look that up in a book, you don’t have to go to (this) University to learn that.’” |
